# Identification and functional analysis of the *LEAFY* gene in longan flower induction

**DOI:** 10.1186/s12864-024-10229-x

**Published:** 2024-03-25

**Authors:** Dengwei Jue, Zhexin Li, Wenlin Zhang, Jianmin Tang, Ting Xie, Xuelian Sang, Qigao Guo

**Affiliations:** 1 Chongqing Key Laboratory of Economic Plant Biotechnology, Collaborative Innovation Center of Special Plant Industry in Chongqing, Chongqing Engineering Research Center for Special Plant Seedling, Institute of Special Plants, Chongqing University of Arts and Sciences, 402160 Yongchuan, China; 2 Key Laboratory of Horticulture Science for Southern Mountains Regions of Ministry of Education, College of Horticulture and Landscape Architecture, Southwest University, 400715 Chongqing, Beibei China

**Keywords:** Longan (*Dimocarpus longan*), *LEAFY* gene, DAP-Seq, Functional identification

## Abstract

**Background:**

Flowering at the right time is a very important factor affecting the stable annual yield of longan. However, a lack of knowledge of the regulatory mechanism and key genes of longan flowering restricts healthy development of the longan industry. Therefore, identifying relevant genes and analysing their regulatory mechanism are essential for scientific research and longan industry development.

**Results:**

*DlLFY* (*Dimocarpus longan LEAFY*) contains a 1167 bp open reading frame and encodes 388 amino acids. The amino acid sequence has a typical LFY/FLO family domain. *DlLFY* was expressed in all tissues tested, except for the leaf, pericarp, and pulp, with the highest expression occurring in flower buds. Expression of *DlLFY* was significantly upregulated at the early flower induction stage in “SX” (“Shixia”). The results of subcellular localization and transactivation analysis showed that DlLFY is a typical transcription factor acting as a transcriptional activator. Moreover, overexpression of *DlLFY* in *Arabidopsis* promoted early flowering and restrained growth, resulting in reduced plant height and rosette leaf number and area in transgenic plants. DNA affinity purification sequencing (DAP-Seq) analysis showed that 13 flower-related genes corresponding to five homologous genes of *Arabidopsis* may have binding sites and be putative target genes. Among these five flower-related genes, only *AtTFL1* (*terminal flower 1*) was strongly inhibited in transgenic lines.

**Conclusion:**

Taken together, these results indicate that *DlLFY* plays a pivotal role in controlling longan flowering, possibly by interacting with TFL1.

**Supplementary Information:**

The online version contains supplementary material available at 10.1186/s12864-024-10229-x.

## Background

As a subtropical perennial crop widely planted in southern China, longan is best known for its nutritious fruit, which has relatively high medicinal value [1]. Flowering at the right time is a very important factor affecting stable annual crop yield and healthy development of the longan industry. However, there are many environmental conditions that can trigger irregular flowering of longan, such as frost in spring and high temperature and moisture in winter [2, 3]. By using chemical treatments, such as potassium chlorate (KClO_3_), to regulate longan tree flowering time, a stable high yield can be obtained [4]. However, the promoting effect is greatly affected by the application area and variety [5]. The key to solving this problem is to analyse the regulatory mechanism of longan flowering.

In recent decades, substantial progress has been made in understanding the physiological and molecular mechanisms underlying flowering time in plants, especially in *Arabidopsis*. The molecular mechanism of *Arabidopsis* flowering is a complex gene regulatory network comprising at least six flowering pathways and several flowering-related genes, such as *flowering locus T* (*FT*), *CONSTANS* (*CO*), *flowering locus C* (*FLC*), and *LFY* [6]. Among these integral genes, *LFY*, a floral meristem identity gene, is a master regulator of flower initiation in *Arabidopsis* [7]. LFY is a plant-specific transcription factor that regulates floral meristem and organ formation by binding target genes, including *APETALA1* (*AP1*), *APETALA3* (*AP3*), *CAULIFLOWER* (*CAL*), *AGAMOUS* (*AG*), *SEPALLATA* (*SEP*), and *TFL1* [8, 9]. Since the first *LFY* gene was identified in *Antirrhinum majus* [10], *LFY* homologous genes have been identified in several woody plants, including apple [11], citrus [12], Fig. [13], and mango [14]. LFY proteins, which contain conserved C-terminal and N-terminal regions, are highly conserved across plant species. However, their function may differ among species. The *LFY* gene of most plants promotes plant flowering [13]. For example, in *Arabidopsis*, overexpression of *LFY* induces early flowering [15], whereas the *Arabidopsis lfy* mutant shows late flowering [7]. Similar situations can be found in many plants, including rice (*Oryza sativa* L.) [16], citrus (*Citrus reticulata* Blanco) [12], poplar (*Populus tomentosa*) [17], fig (*Ficus carica* L.) [13], and mango (*Mangifera indica* L.) [14]. Opposite results have also been reported; for example, overexpression of the tobacco *LFY* homologue *NFL1* in *Arabidopsis* does not severely affect flowering [18]. Therefore, it is necessary to further study the function of *LFY* homologues in different species.

Zeng et al. [19] identified a *LFY* homologue (*LLFY*) from the “Honghezi” longan tree. RT‒PCR results suggest that *LLFY* may be involved in inflorescence differentiation and maintenance in longan. Furthermore, we reported in our previous study that the longan *LFY* gene showed significant upregulation during early flower induction in “SX” (“Shixia”) [5]. These results indicate that the longan *LFY* gene might be involved in flower induction, though its function and regulatory mechanism during flowering are still unknown. Thus, the aim of the present study was to analyse these unknown factors and provide useful information for analysing the regulatory mechanism of longan flowering.

## Results

### Isolation of the *LEAFY* gene of longan

**Fig. 1:**
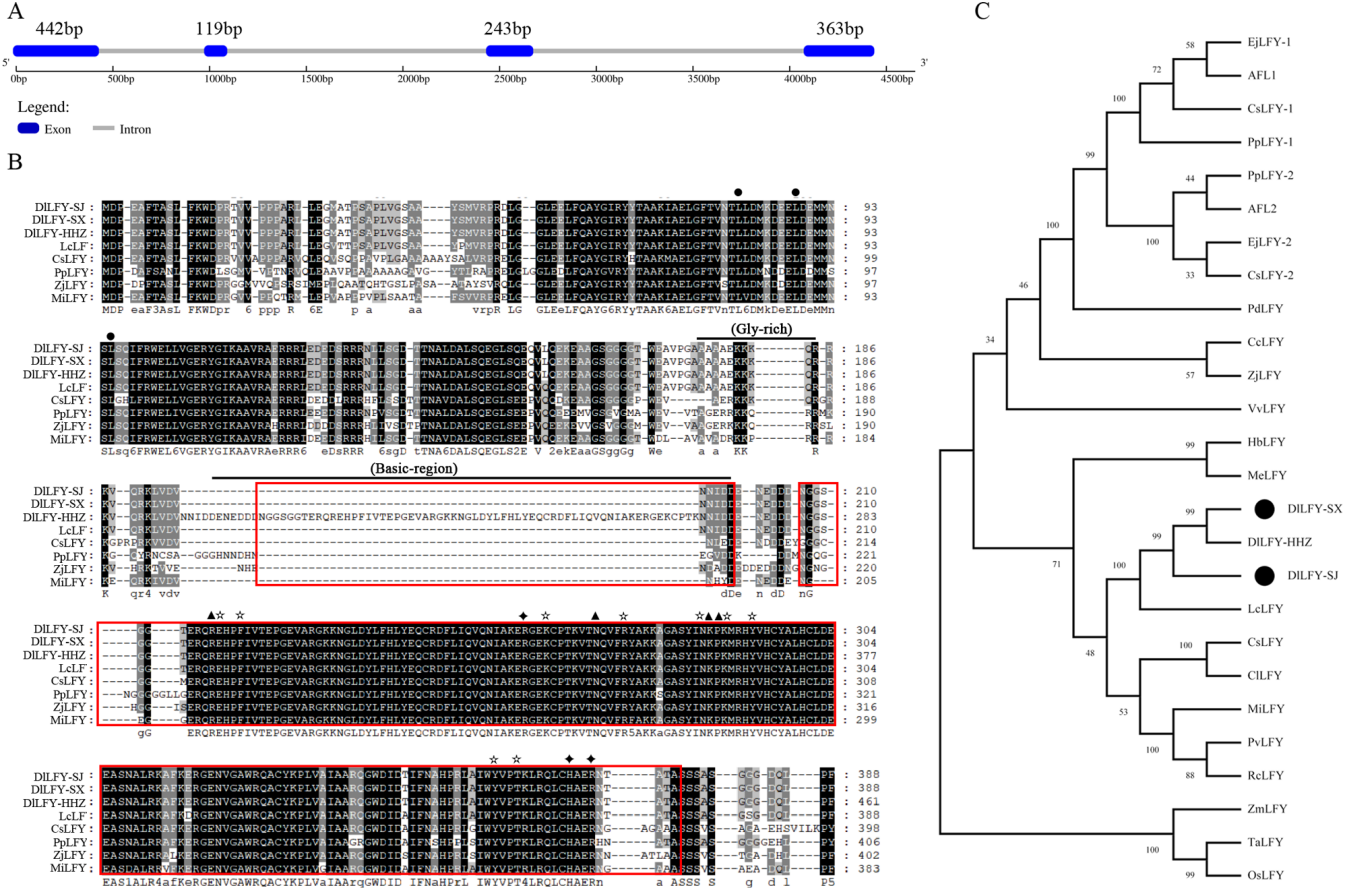
Sequence analysis of *LFY* genes. (**A**) Gene structure of the *DlLFY*-SJ gene. (**B**) Amino acid sequence alignment of LFY proteins from different species. The red box represents FLO/LFY domains, ●represents leucine residues in the leucine repeat domain, ▲represents residues involved in interactions with DNA bases, ✫represents residues involved in interactions with DNA backbones, and ✦represents residues involved in dimerization in the conserved C-terminus. (**C**) Phylogenetic tree of LFY proteins from various species. Gene names, species, and GenBank accession numbers are as follows: DlLFY-HHZ (*Dimocarpus longan* “Honghezi”, Dlo_005438.1), LcLFY (*Litchi chinensis*, AGR45584.1), CsLFY (*Citrus sinensis*, AAR01229.1), CcLFY (*Carya cathayensis*, ABI58284.1), PpLFY-1 (*Pyrus pyrifolia*, BAD10950.1), PpLFY-2 (*Pyrus pyrifolia*, BAD10956.1), ZjLFY (*Ziziphus jujuba*, AEK70963.2), VvLFY (*Vitis vinifera*, XP_002284664.1), EjLFY-1 (*Eriobotrya japonica*, AB162033.1), EjLFY-2 (*Eriobotrya japonica*, AB162039.1), AFL1 (*Malus domestica*, BAD10949.1), AFL2 (*Malus domestica*, BAB83097.1), CsLFY-1 (*Chaenomeles sinensis*, BAD10953.1), CsLFY-2 (*Chaenomeles sinensis*, BAD10959.1), PdLFY (*Prunus dulcis*, AAY30859.1), ClLFY (*Clausena lansium*, ABF61861.2), HbLFY (Hevea brasiliensis, XP_021673364.1), PvLFY (Pistacia vera, XP_031277338.1), RcLFY (Rhus chinensis, AGW47920.1), MeLFY (Manihot esculenta, XP_021615449.1), TaLFY (*Triticum aestivum*, BAE78663.1), OsLFY (*Oryza sativa*, AHX83809.1), ZmLFY (*Zea mays*, ABC69153.1), and MiLFY (*Mangifera indica*, ADX97315.1)

We cloned the *DlLFY* gene from “SJ” (“Sijimi”) and “SX” longan leaf tissues and named them *DlLFY*-SJ and *DlLFY*-SX. Interestingly, the base sequence and amino acid sequence of *DlLFY*-SJ and *DlLFY*-SX are identical but differ from those of DlLFY-HHZ (“Honghezi”) (Dlo_005438.1) (Fig. S1). The full-length DNA sequence of *DlLFY*-SJ and *DlLFY*-SX is 4430 bp, comprising four exons and three introns (Fig. 1A). The cDNA length of *DlLFY*-SJ and *DlLFY*-SX is 1167 bp, encoding 388 amino acid proteins. The predicted protein molecular weight is 43.34 kDa, and the isoelectric point is 7.15. The N- and C-terminal regions of DlLFY-SJ and DlLFY-SX proteins are highly conserved across species (Fig. 1B). Predictive analysis of the conserved region of DlLFY-SJ and DlLFY-SX protein showed that it contains one typical FLO/LFY domain; DlLFY-HHZ contains two typical FLO/LFY domains (Fig. 1B and S1). DlLFY from the three longan varieties have some conserved structures, including a glycine-rich region in front of the alkaline region, a leucine repeat at the N-terminus, and some conserved sites at the C-terminus, including those for its TF responsible for interaction with related region of the downstream target gene promoter. Multiple sequence alignment analysis showed that sequence identity between DlLFY-SJ and DlLFY-SX and LFY homologous amino acids from multiple species is between 58% and 98%. Among them, the sequence identity of DlLFY-SJ and DlLFY-SX with LcLFY (AGR45584.1), which all belong to Sapindaceae, is highest, at 98%, followed by homology with other dicotyledonous fruit tree LFY proteins, such as CsLFY (*Citrus sinensis*, AAR01229.1), CcLFY (*Carya cathayensis*, ABI58284.1), and PpLFY-1 (*Pyrus pyrifolia*, BAD10950.1). Homology with monocotyledonous plant LFY proteins, including TaLFY (*Triticum aestivum*, BAE78663.1), OsLFY (*Oryza sativa*, AHX83809.1), and ZmLFY (*Zea mays*, ABC69153.1), is lowest (Fig. 1C).

### Analysis of *DlLFY* gene expression in different tissues and flowering development in longan

**Fig. 2:**
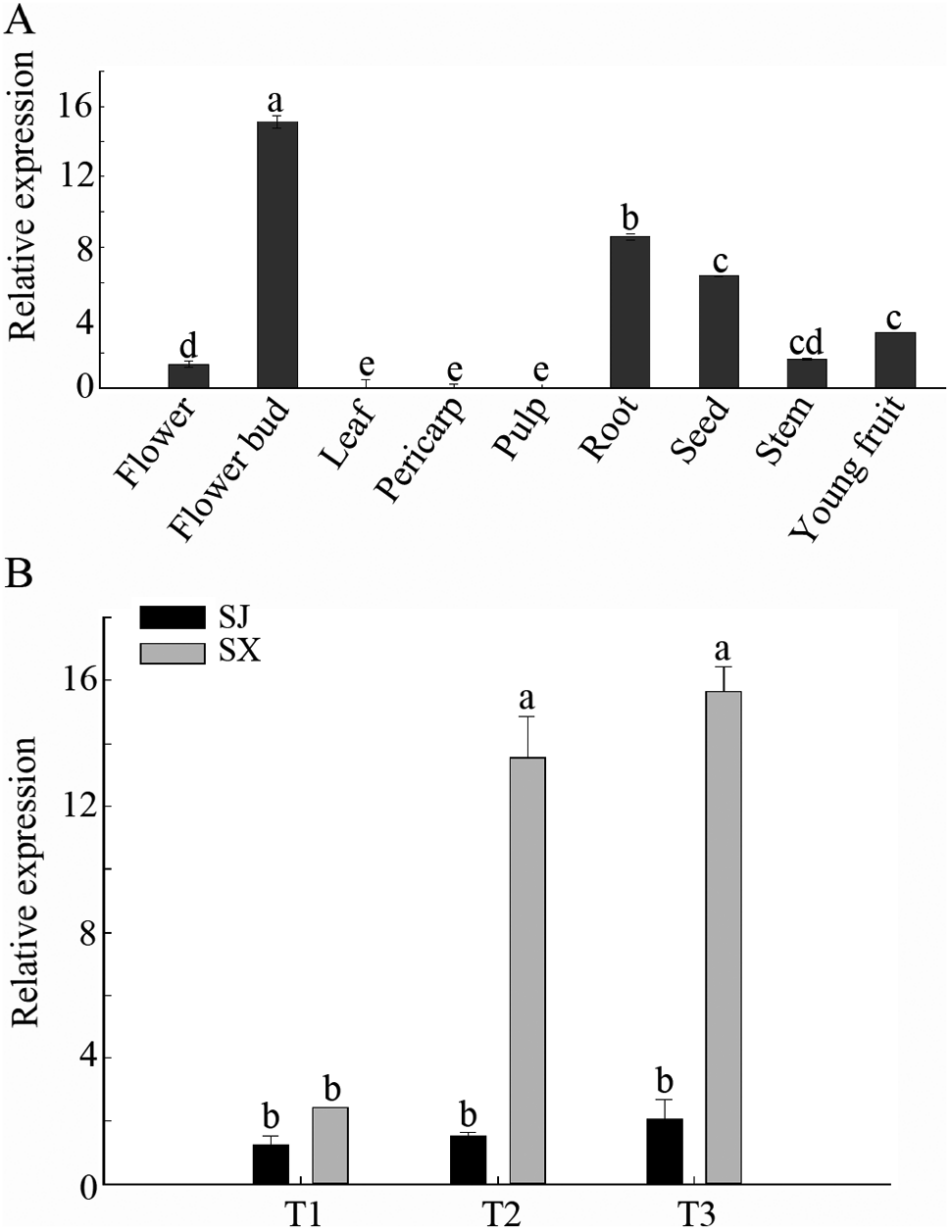
Relative expression levels of *DlLFY* genes. (**A**) Tissue expression patterns of the *DlLFY* gene in the flower, flower bud, leaf, pericarp, pulp, root, seed, stem, and young fruit. (**B**) Expression pattern of the *DlLFY* gene in SX and SJ during different flower development stages. Three different types of SX and SJ apical buds were used in this study. Samples of the dormant stage (before the emergence of floral primordia) (T1) were collected on November 20, 2016; the apical bud at this stage is characterized by high hardness. Samples of floral primordia (red bud) (T2 stage) were collected on December 24, 2016; the apical bud at this stage is characterized by the appearance of red dot. Samples of the floral organ formation stage (T3) were collected on January 1, 2017; the apical bud at this stage is characterized by the appearance of the first inflorescence, Three biological replicates from three different trees were used for each sample. Values with different letters indicate significant differences between samples in SX or SJ with *p* < 0.05

The expression patterns of the *DlLFY* gene in different tissues and the flowering development of longan were analysed by using qRT–PCR. As shown in Fig. 2A, *DlLFY* expression was detected in all tissues tested, except for the leaf, pericarp, and pulp, albeit at different levels. Flower buds showed high *DlLFY* accumulation, followed by roots, seeds, and young fruits. In addition, the expression pattern of *DlLFY* at the early flower induction stage in SX showed obvious upregulation, while it did not significantly change in SJ (Fig. 2B).

### Subcellular localization and transactivation analysis of DlLFY

**Fig. 3:**
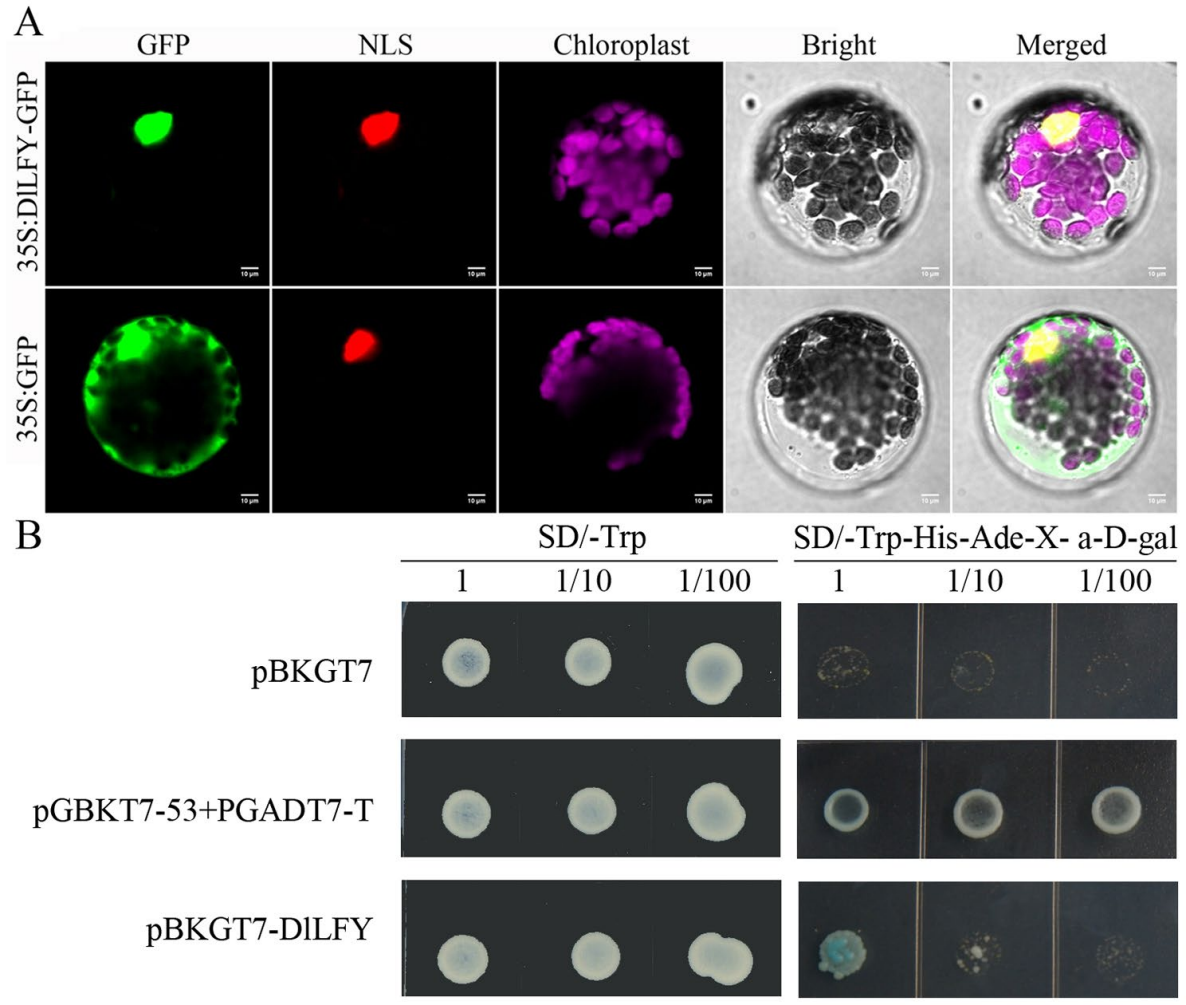
Subcellular localization and transactivation analysis of DlLFY. (**A**) Subcellular localization of the DlLFYGFP fusion protein in *Arabidopsis* protoplasts. The fluorescence signals of GFP (Green fluorescent protein), NLS (Nuclear locating sequence) and Chloroplast in protoplasts were observed at a wavelength of 488 nm, 561 and 640 nm, respectively. Scale bar = 10 μm. (**B**) Transactivation activity analysis of full-length DlLFY in yeast. The initial concentration of yeast was adjusted to an OD_600_ of 0.1 and diluted 1/10, 1/100. Negative control, pGBKT7; positive control, pGBKT7-53 + pGADT7-T

To determine the subcellular localization of the DlLFY protein, the recombinant plasmid pBWA(V)HS-DlLFY-osGFP was generated and introduced into *Arabidopsis* protoplasts. As shown in Fig. 3A, under confocal laser scanning microscopy, the fluorescence signal from the DlLFY-GFP fusion protein was mainly detected in the nucleus, and were colocalized with nuclear locating sequence (NLS), whereas the GFP control was detected in both the nucleus and cytoplasm (Fig. 3A). These results demonstrate that DlLFY is a nuclear protein. To investigate the transcriptional activity of DlLFY, yeast one-hybrid assays were performed. Full-length DlLFY was inserted into the pGBKT7 vector, and the recombinant plasmid, the negative control (pGBKT7), and the positive control (pGBKT7-53 + pGADT7-T) were transformed into yeast strain AH109. All transformed yeast strains grew well on SD/-Trp medium. Yeast strains carrying the positive control vector (pGBKT7-53 + pGADT7-T) and the full-length DlLFY (DlLFY residues 1–388) grew well and appeared blue on SD/Trp-/His-/Ade-/X-α-gal selection medium, whereas cells containing the negative control vector (pGBKT7) did not grow (Fig. 3B). These results indicate that DlLFY is a transcriptional activator.

### DlLFY functions as a positive regulator of plant flowering

**Fig. 4:**
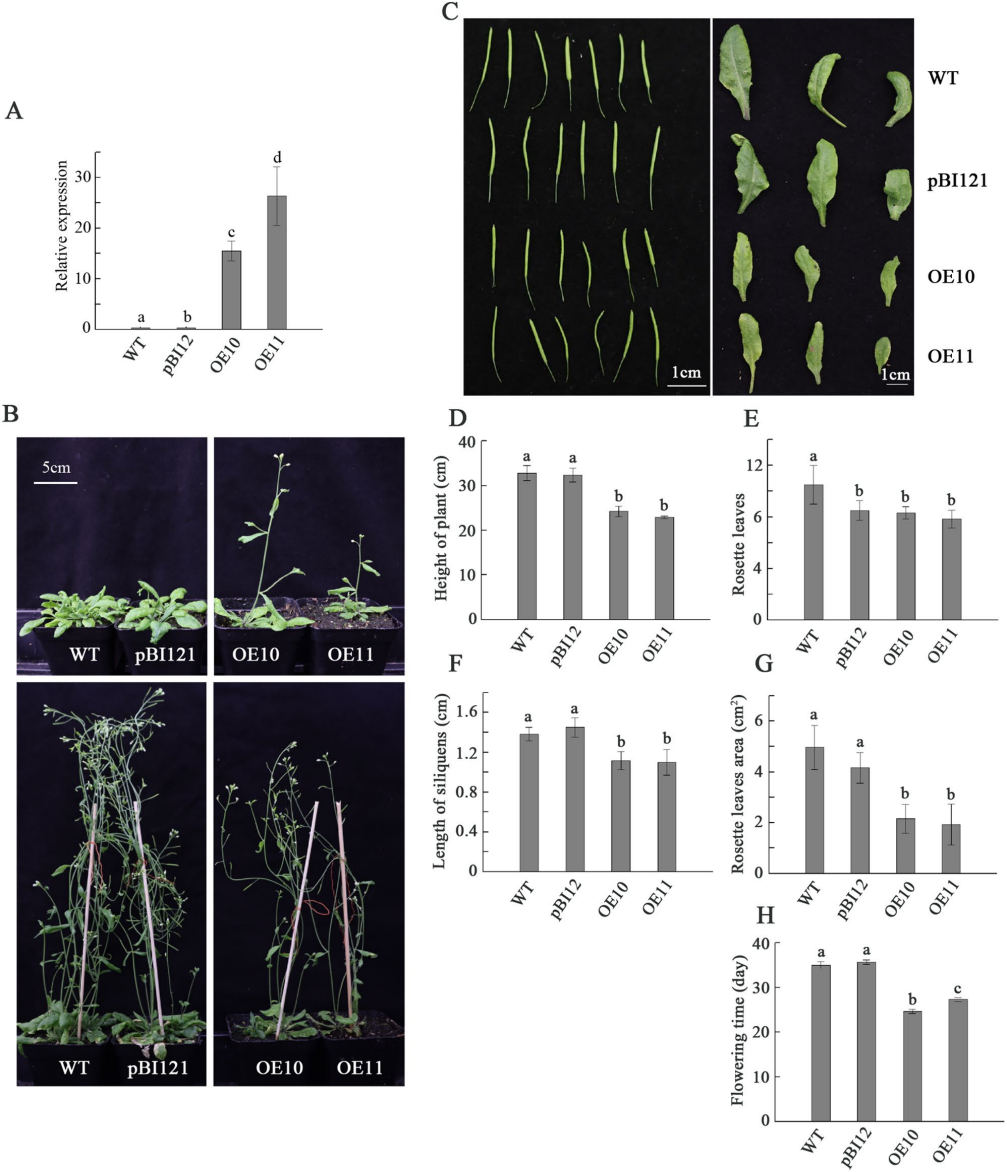
Phenotype analysis of *DlLFY*-overexpressing transgenic plants. (**A**) Detecting expression of *DlLFY* in transgenic plants and controls by qRT‒PCR. Values with different letters indicate significant differences between samples with *p* < 0.05. (**B**) Phenotypes of *DlLFY*-overexpressing, positive control, and negative control *Arabidopsis* at different flowering times (top: 28-day-old plants; bottom: 54-day-old plants). (**C**) Different silique and leaf phenotypes of *DlLFY*-overexpressing, positive control, and negative control *Arabidopsis* plants. (**D**) Comparison of plant height between *DlLFY* transgenic plants and controls. (**E**) Comparison of the number of rosette leaves between *DlLFY* transgenic plants and controls. (**F**) Comparison of silique length between *DlLFY* transgenic plants and controls. (**G**) Comparison of rosette leaf area between *DlLFY* transgenic plants and controls. (**H**) Comparison of flowering time between *DlLFY* transgenic plants and controls. Values are the means of three replicates ± SE. Values with different letters indicate significant differences between samples with *p* < 0.05

To further investigate the function of *DlLFY*, we constructed an overexpression vector and introduced it into *Arabidopsis* (Col-0). Wild-type Col-0 (WT) plants and WT plants transformed with the pBI121 empty vector were used as negative and positive controls, respectively. A total of 15 independent transgenic lines were obtained, and two homozygous T3 transgenic lines were randomly selected for further phenotypic analysis. The expression level of the exogenous *DlLFY* gene in transgenic plants was detected by qRT‒PCR. The specific primers used for the *DlLFY* gene were designed based on sequence alignment between *DlLFY* and *AtLFY* (Table S1). The results showed that *DlLFY* was overexpressed in the transgenic *Arabidopsis thaliana* but not in control plants (Fig. 4A). Compared with control plants, the growth of *Arabidopsis* plants overexpressing *DlLFY* was restrained (Fig. 4B, C). The average height of the transgenic plants was between 22.96 (OE11) and 24.30 (OE10) cm, which was significantly shorter than that of the WT (32.41 cm) and pBI121 lines (32.80 cm) (Fig. 4B, D). The average number of rosette leaves in WT plants was 10.5, which was significantly higher than that in transgenic plants (between 7.8 and 8.3). There were no significant differences between the pBI121 (8.5) line and the transgenic plants (Fig. 4B, E). The transgenic plants also had smaller siliques and rosette leaf areas. The average length of siliques of OE10 and OE11 was 1.12 and 1.1 cm, respectively, compared to 1.38 and 1.45 cm for the negative and positive control lines, respectively (Fig. 4F). As shown in Fig. 4G, the rosette leaf area was reduced to 2.81 and 2.97 cm^2^ in OE10 and OE11, respectively, compared with WT plants. Interestingly, although overexpression of the *DlLFY* gene may restrain the growth of *Arabidopsis* plants, our results show that transgenic plants flowered earlier than control plants (Fig. 4B, H). The flowering time ranged from 24.67 to 27.33 d for transgenic plants and from 31.67 to 35 d for control plants (Fig. 4B, H).

### DAP-Seq analysis

To analyse the mechanism of *DlLFY* gene regulation of plant flowering, DAP-Seq analysis was performed. First, the *DlLFY* CDS was cloned, verified, and subcloned and inserted into the pFN19K HaloTag^@^ T7 SP6 Flexi Vector (Promega #G184A) to express the recombinant protein. The clones were mixed with the “SJ” T2 flower bud DNA library to identify putative genes.

**Fig. 5:**
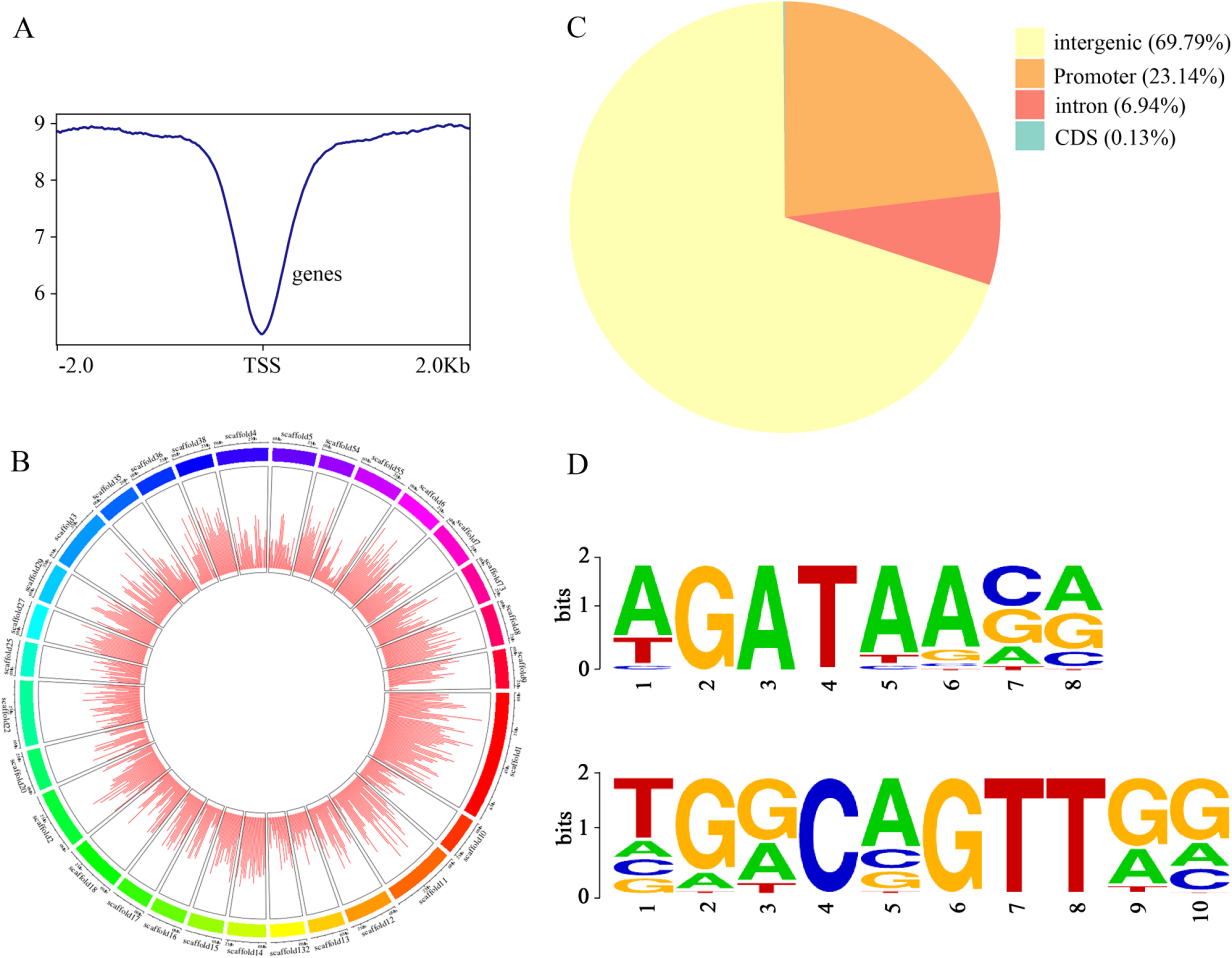
DAP-Seq signal, peak distribution, and motif information. (**A**) DAP-Seq signal upstream or downstream of the 2 k TSS interval. (**B**) Genomic distribution of peaks: 69.79, 23.14, 6.94, and 0.13% of peaks are separately located in intergenic, promoter, intron, and CDS regions, respectively. (**C**) Peak distribution on different chromosomes. Chromosomes 10 and 100 account for the top two groups. (**D**) Top two motifs identified by MEME-ChIP

In total, 4.26 × 10^7^ clean reads were obtained, and 98.02% of reads were mapped to the *D*. *longan* genome (Table S2). Then, 3.87 × 10^7^ unique mapped reads were used for data analysis. Based on peak calling with MACS2 software, 75,346 peaks were identified (q < 0.05), with an average length of 530 bp (Fig. 5A). Scaffold 1, scaffold 10, and scaffold 100, containing 1720, 263 and 209 peaks, respectively, were the top three scaffolds (Fig. 5B). Genomic region analysis showed that 69.79, 23.14, 6.94, and 0.13% of the peaks were separately located in the intergenic, promoter, intron, and CDS regions, respectively (Fig. 5C). Gene annotation for peaks identified 25,098 single genes (Table S3). The results of GO function enrichment showed that among these 25,098 genes, 9835 genes were significantly enriched in DNA-binding transcription factor activity, transcription regulator activity, regulation of RNA biosynthetic process, regulation of transcription, and DNA binding GO terms (q-value < 0.05) (Fig. S2A and Table S4). KEGG pathway enrichment analysis showed that the MAPK signalling pathway (q-value = 1.08 × 10^− 3^, 187 genes), phenylpropanoid biosynthesis (q-value = 1.89 × 10^− 3^, 221 genes), glycerolipid metabolism (q-value = 2.41 × 10^− 3^, 95 genes), plant hormone signal transduction (q-value = 7.23 × 10^− 3^, 332 genes), beta-alanine metabolism (q-value = 1.96 × 10^− 2^, 56 genes), purine metabolism (q-value = 3.05 × 10^− 2^, 115 genes), and pentose and glucuronate interconversion (q-value = 3.41 × 10^− 2^, 198 genes) were enriched (Fig. S2B and Table S5).

Moreover, motif analysis identified 82 enriched motifs (q-value < 0.05). The top two significant motif sequences, MEME-1_ AGATAASR and MEME-2_TGGCAGTTGG, are shown in Fig. 5D and Table S6.

### Identification of the target flower-related genes of *DlLFY*

**Fig. 6:**
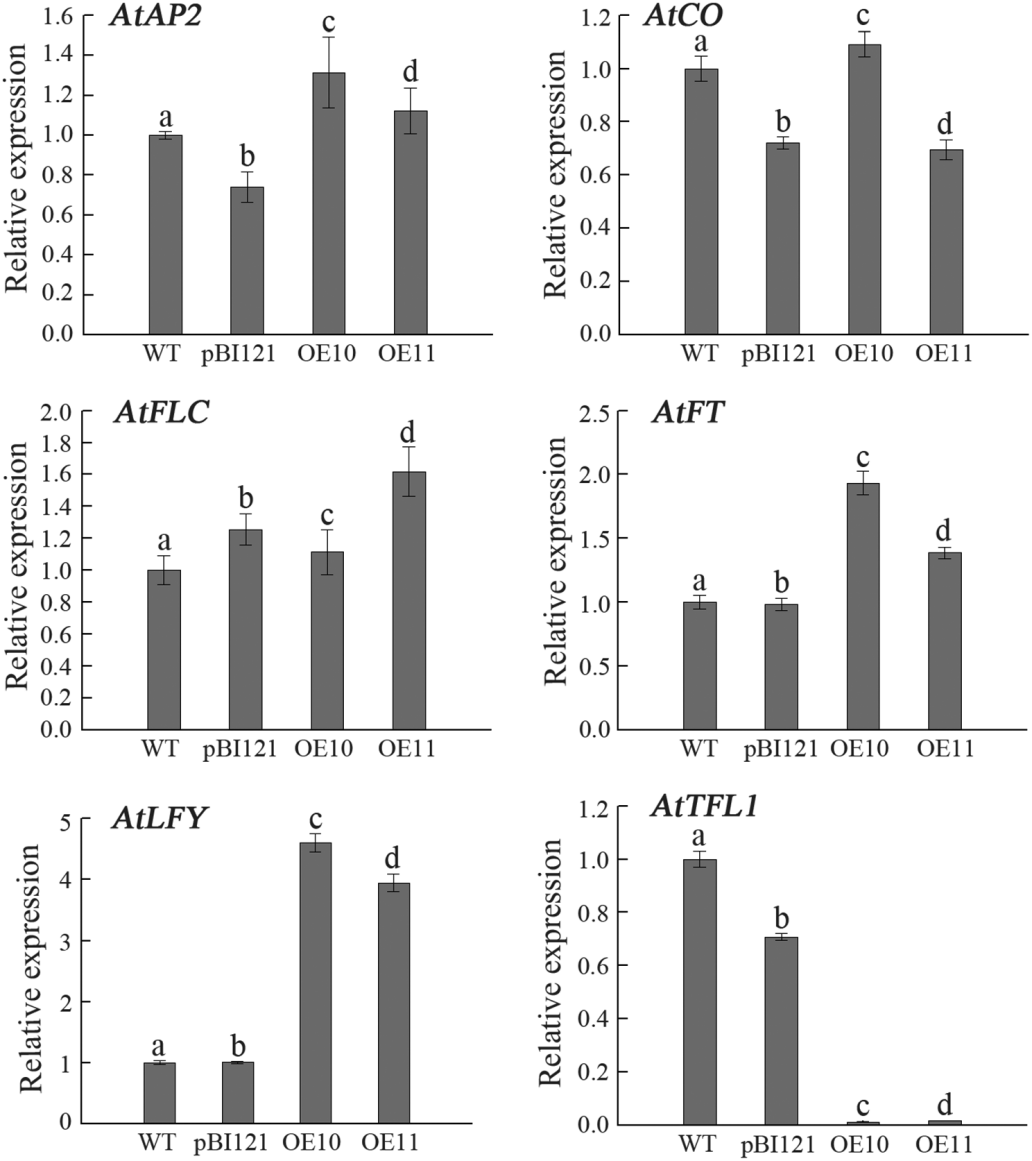
Expression of flowering-related genes *AtAP2*, *AtCO*, *AtFLC*, *AtFT*, *AtLFY*, and *AtTFL1* in transgenic and control lines verified by qRT–PCR. RNA samples were extracted from 35-day-old seedlings, which were grown in pots under LD conditions. Transcript levels were normalized using *AtActin2* as a reference gene. Values with the same letter were not significantly different with *p* < 0.05 (*n* = 3)

As a transcription factor, LFY mainly regulates plant flowering by activating or inhibiting other flower-related genes. Among the 25,098 single genes, we found 13 flower-related genes that might be regulation targets of DlLFY: one *TFL1* gene (Dlo_032530.1), four *FT* genes (*flowering locus T-like 2*, Dlo_012579.1; *flowering locus T-like* 3, Dlo_000296.1; *flowering locus T2*, Dlo_014365.1; *FLOWERING LOCUS T2*, Dlo_012576.1), one *FLC* gene (Dlo_031929.1), six *CO-like* genes (*CONSTANS-LIKE 4*, dlo_035278.1; *CONSTANS-like protein*, Dlo_003684.1; *CONSTANS-LIKE 14-like*, Dlo_031781.1; *CONSTANS-LIKE 14-like*, Dlo_029236.3; *CONSTANS-LIKE 10*, Dlo_013961.1; *CONSTANS-LIKE 7-like*, Dlo_005461.1), and one *AP2* gene (Dlo_000287.1) (Table S7). To identify the relationship between these genes and *LFY*, qRT‒PCR was used to detect the transcription levels of the target flower-related homologous genes in negative and positive controls and overexpression lines. As shown in Fig. 6, among these five genes, the expression level of *AtTFL1* was strongly inhibited in the two overexpression lines, whereas the other four genes did not show significant changes. The expression levels of *AtTFL1* in OE10 and OE11 were 1/83 and 1/67 of that in the control, respectively. Only one peak (IP_SJ_vs_In_SJ_peak_71679) was for the *TFL1* gene (Dlo_032530.1), with the motif AMYB (HTH), which is one of the two top motifs in this study (Table S7). These results suggest that the relationship between LFY and TFL1 may be important to coordinate flowering responses which needs further confirmation and analysis.

## Discussion

The *LFY* gene encodes a plant-specific DNA-binding transcription factor that has important roles in flowering pathway regulation. The FLO/LFY domain, which has been proven to be involved in the development of inflorescence and floral meristem formation in *Arabidopsis*, is highly conserved among plant species [13, 20]. In the present study, consistent with LFY proteins in other plants [20], DlLFY from the “SJ” and “SX” longan varieties (DlLFY-SJ and DlLFY-SX) only contain one typical FLO/LFY domain. However, DlLFY-HHZ contains two typical FLO/LFY domains. Among these three longan varieties, “SX” and “Honghezi” have the SF trait of flowering and bearing fruit once each year. However, “SJ” has the PF trait of flowering and bearing fruit throughout the whole year and does not need appropriate environmental factors [5, 21]. Therefore, the number of FLO/LFY domains may not be the key factor by which LFY acts as a TF during flowering regulation in longan.

The expression level of genes can reflect their role in the development of plant organs and tissues. Previous studies have reported that *LFY* shows diverse expression in various organs and tissues of plants. Accumulated data show that the *LFY* gene is mainly expressed in flower organs, especially in the early developmental stage of flower induction [13]. For example, *JcLFY* (*Jatropha curcas* L.) is expressed in inflorescence buds, flower buds, and carpels, with the highest expression occurring in the early development stage of flower buds [15]. *MiLFY* is more highly expressed in the flowers and stems of flowering branches than in those of nonflowering branches and correlates with the floral development stage in different cultivars [14]. *LcLFY* is mainly expressed in flower buds but barely detectable in stems, mature leaves, petioles, and pedicels [22]. Consistent with those studies, we found that *DlLFY* expression was highest in flower buds, followed by roots, seeds, and young fruits, whereas we did not detect DlLFY in leaves, pericarp, or pulp, indicating that *DlLFY* may mainly participate in the development of flower buds. In addition, expression of *DlLFY* was significantly upregulated at the early flower induction stage in “SX” but did not show a significant change in “SJ”. These results indicate that the *DlLFY* gene may mainly act in early flower induction and play different roles in different plant varieties.

Previous studies have shown that the correlation between transcript abundance and protein concentration is poor due to translation regulation [23], which means that the expression level of a gene may not truly reflect its biological function. Thus, transgenes are the most direct and effective method to study the biological function of genes. Since longan has no regeneration system, we conducted genetic transformation of *Arabidopsis thaliana* to verify the function of the *DlLFY* gene. Many studies have shown that ectopic expression of *LFY* genes in different plants results in early flowering. For example, transgenic *Arabidopsis* lines overexpressing the *FcLFY* gene flower approximately 6 and 8 days earlier than WT or the corresponding mutant *lfy-15* [13]. Overexpression of *VpLFY2* causes precocious flowering in *Arabidopsis* [24]. Similar results were also found in *Brassica juncea* [25], jatropha [15], and mango [14]. Consistent with those studies, the 35 S:*DlLFY* transgenic *Arabidopsis* lines OE10 and OE11 in the present study flowered approximately 4 and 7 days earlier than WT. These results indicate that the function of the *LFY* gene in promoting flowering is conserved in different plants. Interestingly, in addition to the flowering-promoting phenotype, some morphological variations were observed in the transgenic lines, showing that overexpression of the *DlLFY* gene restrains the growth of *Arabi*dopsis plants. For example, the transgenic plants were shorter, with fewer rosette leaves and smaller siliques and rosette leaf areas, than the control plants. Similarly, lines with overexpression of *FcLFY* show fewer rosette and cauline leaves and shorter plant heights. However, there were more rosette leaves on transgenic C7 than WT plants, and our results were different [13]. Overexpression of the *AfLFY* gene in tobacco promotes precocious flowering with obvious changes in leaf shape among transgenic lines [26]. Overexpression of *MiLFY* promotes early flowering in transgenic *Arabidopsis* lines, with decreased flower petal number and some short or curved pods [14]. These results suggest that the *DlLFY* gene can inhibit the vegetative growth of plants by shortening the vegetative growth period, similar to homologous genes of other plants, though the inhibition phenotypes may differ among plants.

LFY is a plant-specific transcription factor localized to the nucleus [14, 26]. In the present study, subcellular localization and transactivation analysis showed that DlLFY is a typical transcription factor. Thus, we sought to determine how it promotes plant flowering, by promoting expression of floral organ-specific identity genes such as AG, and AP3 or by interacting with flowering inhibitor genes such as TFL1 and FLC or both.

The first step to address these questions is to identify binding sites and putative target genes. Chromatin immunoprecipitation (ChIP) is currently the leading method for identifying direct regulatory targets of TFs [27]. However, gene-specific antibodies or tagged transgenic lines need to be created for this method, which can be technically challenging and expensive, limiting its use [28]. In contrast to ChIP, DAP-Seq does not need sample-specific reagents such as antibodies or gene-specific primers, making it faster and less expensive, with easier identification of direct regulatory targets of TFs [27]. Indeed, DAP-Seq has proven to be a powerful tool for determining genome-wide binding sites of TFs in plants, especially fruit trees [29, 30]. In the present study, our DAP-Seq analysis detected 75,346 TF-binding events assigned to 25,098 single genes. Among those 25,098 genes, 9835 genes were significantly enriched in DNA-binding transcription factor activity or DNA binding GO terms. Similar results were also found in other studies [31, 32]. LFY is a master regulator in the complex floral gene network [7]. *LFY* can determine the plant’s flower initiation by activating downstream floral meristem identity genes such as *AP1* and *CAL* [33, 34] or inhibiting downstream flowering inhibitor genes such as *TFL1* and *FLC* [9, 13]. In this study, we found 13 flower-related genes corresponding to 5 homologous genes of *Arabidopsis*. Among these five flower-related genes, only *AtTFL1*, containing the top motifs (AMYB, HTH), was strongly inhibited in *DlLFY*-overexpressing transgenic lines. Consistent with our results, expression levels of *AtTFL1* were decreased in *FcLFY* overexpression lines [13]. Our results suggest that *DlLFY* might promote flowering by interacting with TFL1.

## Materials and methods

### Plant materials

The longan cultivars “SJ” and “SX” were grown in the same orchard in Mazhang district (110°16′ E, 21°10′ N), Zhanjiang, Guangdong Province, P.R. China. “SJ” is a perpetual flowering (PF) genotype that has the ability to flower and bear fruit throughout the whole year and does not need the appropriate environmental factors. “SX” is a typical seasonal flowering (SF) longan cultivar, which means that appropriate environmental factors, such as an appropriate period of low temperatures to accumulate energy and nutrients for flower induction, are necessary for flowering [5]. For a common longan cultivar, such as “SX”, flower induction will occur from December of the year to January of the next year. Three developmental flower bud samples of these two cultivars, including dormant apical bud (T1), floral primordia differentiation (red bud) (T2), and floral organ formation (T3), were obtained on 20 November 2016, 24 December 2016, and 1 January 2017. All samples were collected from 10:00 am to 12:00 am, frozen immediately in liquid nitrogen and stored at ‒80 °C. Among the three flower bud samples, the sample of stage T2 of “SJ” was used for DAP-Seq analysis. For tissue expression analysis, nine “SJ” longan tissues (flower, flower bud, leaf, pericarp, pulp, root, seed, stem, and young fruit) were collected from November 2016 to April 2017. The *Arabidopsis* ecotype Columbia (Col-0) plants used for transformation were maintained in our laboratory.

### RNA extraction and *DlLFY* gene cloning

Total RNA was extracted from different longan tissues or WT and transgenic *Arabidopsis* lines by using a Quick-RNA isolation kit (Huangyueyang, Beijing, China) according to the manufacturer’s instructions. The RNA quality was detected as described in our previous study [5]. PrimeScript First-Strand cDNA Synthesis Kit (TaKaRa Biotechnology Co., Ltd., Dalian, China) was used for cDNA synthesis. The primers for *DlLFY* gene cloning were designed by using primer 5.0 according to the sequence of Dlo_005438.1 [21].

### Sequence alignment and bioinformatics analysis

BioXM 2.6 software (http://cbi.njau.edu.cn/BioXM/; 01 August 2022) was used to calculate the MW, number of amino acids, ORF, ORF length, and isoelectric point (pI) of the DlLFY protein. The exon–intron structure was analysed by GSDS version 2.0 [35]. Conserved protein domains of the DlLFY protein were predicted with the NCBI Conserved Domain Database online tool (http://www.ncbi.nlm.nih.gov/Structure/cdd/cdd.shtml/; 20 August 2022). Multiple sequence alignment of the LFY protein was performed by using Clustal X version 1.83. Based on this alignment, a phylogenetic tree was constructed by using the neighbour-joining (NJ) method with MEGA 11, with 1000 bootstrap replicates [36].

### Subcellular localization and transcriptional activation activity analysis of DlLFY

The full coding DNA sequence (CDS) of the *DlLFY* gene without the termination codon was amplified using primers SUDlLFY-S and SUDlLFY-A (Table S1). Nuclear locating sequence (NLS, MDPKKKRKV) was used as a nuclear marker and constructed into the pAN580-NLS-mkate vector [37]. Plasmids pBWA(V)HS-osgfp (negative) and pBWA(V)HS-*DlLFY*-osgfp were constructed and separately introduced into *Agrobacterium tumefaciens* strain GV3101 with pAN580-NLS-mkate vector. The pBWA(V)HS-osgfp and pBWA(V)HS-*DlLFY*-osgfp plasmids were transformed into *Arabidopsis thaliana* protoplasts by the PEG-mediated method [38]. After incubation in the dark at 28 ℃ for 24–48 h, the fluorescence signal was observed using a confocal scanning microscope (LSM880; Carl Zeiss, Oberkochen, Germany).

To analyse the transcriptional activation activity of DlLFY, its full-length CDS was cloned and inserted into the pGBKT7 vector with Matchmaker Gold Yeast One-Hybrid System (Clontech, Dalian, China) according to the manufacturer’s instructions. The initial concentration of yeast was adjusted to an OD_600_ of 0.1 and diluted 1/10, 1/100, the empty pGBKT7 vector was used as a negative control, and the interaction between pGBKT7-53 and pGADT7-T served as a positive control. These constructs were transformed into the yeast strain AH109 and subjected to selection on SD/Trp-/His-/Ade-/X-α-gal medium plates at 30 °C for 3–5 days.

### Plant expression vector construction and transformation

The full-length CDS of *DlLFY* was cloned and inserted into the BamHΙ and SacΙ sites in pBI121 under the control of the CaMV35S promoter to construct the overexpression vector. Then, the pBI121-*DlLFY* and pBI121 constructs were introduced into *Agrobacterium* strain GV3101 for *Arabidopsis* transformation using the floral dip method [39]. Seeds were collected and sown on Murashige and Skoog (MS) agar medium containing 25 µg·mL^− 1^ hygromycin for selection under 16 h light/8 h dark conditions at 22 ± 2 °C. Fifteen transgenic plants overexpressing *DlLFY* were obtained and confirmed by β-glucuronidase (GUS) staining of seedlings and RT‒PCR analysis. Two T3 generation homozygous lines (OE10 and 11) were used for further functional analysis. The flowering time, number of rosette leaves, rosette leaf area, length of siliques, and plant height of the transgenic, WT, and empty vector-transformed *Arabidopsis* plants (which were used as controls) were recorded or measured under long-day (LD) conditions.

### Quantitative real-time PCR

Quantitative real-time PCR was performed by using LightCycler® 480 Real-Time PCR System (Roche, Germany) and SYBR Green II PCR Master Mix (Takara, Dalian, China). The amplification programme was performed as described in our previous study [40]. Three biological replicates were carried out for each sample. Transcript levels were calculated using the 2^–∆∆Ct^ method and normalized using the longan *Actin* gene (Dlo_028674) [5] or *Arabidopsis Actin2* as an internal control. Genes that were up- or downregulated by at least twofold were considered differentially expressed.

### DAP-Seq

DAP-Seq was performed as previously described [41]. First, a DAP-Seq genomic DNA library (gDNA of “SJ” T2 flower bud) was prepared by attaching a short DNA sequencing adaptor to purified and fragmented gDNA. The DAP gDNA library was prepared using a NEBNext® DNA Library Prep Master Mix Set for Illumina® (NEB, #E6040S/L). *DlLFY* was fused to HaloTag using a kit from pFN19K HaloTag®T7 SP6 Flexi® Vector (Promega #G184A). *DlLFY* fused to HaloTag was expressed using a TnT®SP6 High-Yield Wheat Germ Protein Expression System (L3260, Promega). Magne HaloTag Beads and the *DlLFY*-HaloTag mixture were incubated with 500 ng DNA library in 40 µL PBS with slow rotation for 1 h at room temperature. The beads were washed 5 times with 200 µL PBS + NP40 (0.005%) and resuspended in PBS. The supernatant was removed, 25 µL Buffer EB was added, and the mixture was incubated for 10 min at 98 °C to elute the bound DNA from the beads. The correct DAP-Seq library concentration to achieve a specific read count was calculated based on library fragment size. The input was the directly obtained genome-wide DNA library. The correct DAP-Seq and input libraries were sequenced using an Illumina NovaSeq 6000 with the PE 150 method.

Trimmomatic (version 0.38) was used to filter out low-quality reads. Clean reads were mapped to the longan genome [21] by BWA (version 0.7.15), allowing up to two mismatches. SAMtools (version 1.3.1) was used to remove potential PCR duplicates, and MACS2 software (version 2.1.1.20160309) was used to call peaks by default parameters (bandwidth, 300 bp; model fold, 5, 50; q-value < 0.05) [42]. Wig files produced by MACS software were used for data visualization by IGV (version 2.3.91). If the summit of a peak was located close to the TSS of one gene, the peak was assigned to that gene [43]. In addition, all peak-related genes were mapped to GO terms in the Gene Ontology database (http://www.geneontology.org/) and KEGG database (https://www.kegg.jp/) [44]. The p value or calculated p value (FDR) ≤ 0.05 was set as the threshold. The MEME suite (http://meme-suite.org/) was used to detect motifs.

### Data analysis

The data were analyzed using the least significant difference method with the SPSS software package, Version 20.0 (IBM Corp., New York, United States) to test significant differences, and the significance level was set at *p* < 0.05.

## Conclusions

In summary, the *LFY* gene, a typical transcription factor demonstrated to be conserved with other FLO/LFY homologue genes, was isolated from longan. *DlLFY* gene transcripts mainly accumulated in flower buds, with significantly upregulation at the early flower induction stage in “SX”. *Arabidopsis* transgenic lines overexpressing the *DlLFY* gene showed early flowering and an inhibited vegetative growth phenotype. DAP-Seq and qRT‒PCR showed that *DlLFY* might promote flowering by interacting with *TFL1*. However, the direct regulatory relationship between TFL1 and DlLFY needs further confirmation and analysis. This study provides useful information for identifying *DlLFY* function during flower induction and will be helpful in accelerating the molecular breeding of longan.

### Electronic supplementary material

Below is the link to the electronic supplementary material.

Supplementary Material 1

Supplementary Material 2

Supplementary Material 3

Supplementary Material 4

Supplementary Material 5

Supplementary Material 6

Supplementary Material 7

Supplementary Material 8

## Data Availability

The “SJ” and “SX” data were uploaded to the NCBI database (http://www.ncbi.nlm.nih.gov/sra) with accession number SRS2241241–SRS2241258. Raw DAP-Seq data were deposited in the NCBI database (http://www.ncbi.nlm.nih.gov/bioproject) with accession number PRJNA867019.
