# Supplementary material for: Identification and functional analysis of the LEAFY gene in longan flower induction
Source: BMC Genomics. 2024 Mar 25;25:308. doi: 10.1186/s12864-024-10229-x (PMC10962150; doi:10.1186/s12864-024-10229-x)
Supplement: Supplementary file 1 — Supplementary Material 1 [file 12864_2024_10229_MOESM1_ESM.docx]

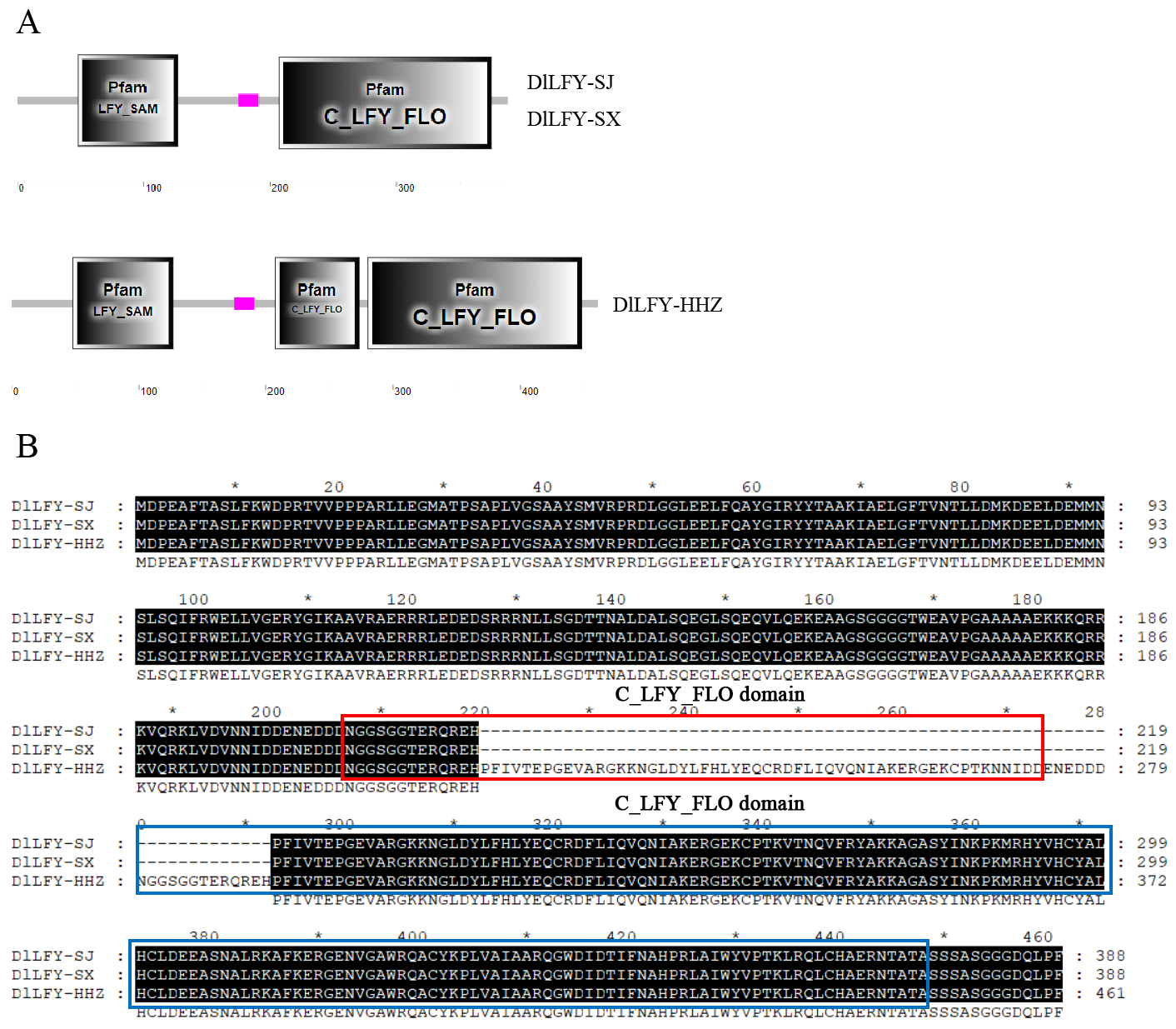


Fig S1. Sequence analysis of LFY genes from different longan varieties. (A) Domain analysis of different DlLFY. (B) Multiple sequence alignment of DlLFY domains. Red and blue boxes represent FLO/LFY domains.


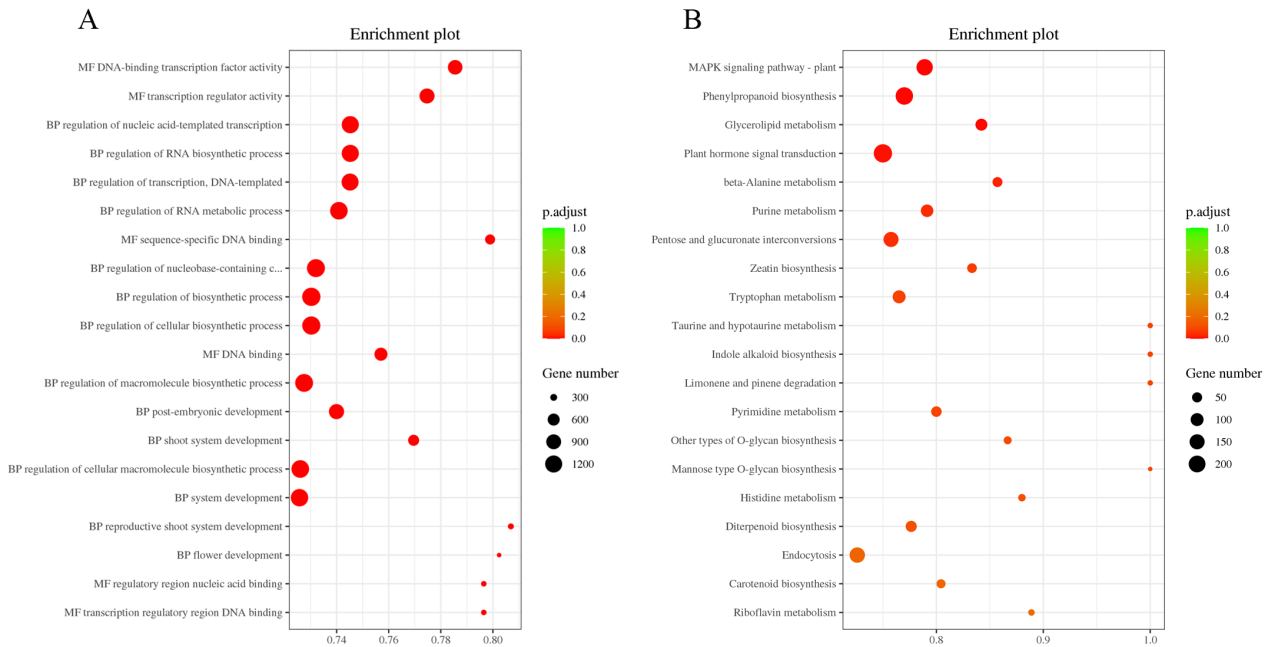


Fig S2. The GO functions enrichment analysis (A) and the KEGG pathway enrichment analysis (B) of DAP-seq analysis.
